# A simple and efficient *Agrobacterium*-mediated *in planta* transformation protocol for horse gram (*Macrotyloma uniflorum* Lam. Verdc.)

**DOI:** 10.1186/s43141-020-00023-z

**Published:** 2020-03-24

**Authors:** Thomas Cheeran Amal, Palanisamy Karthika, Gurusamy Dhandapani, Subramaniam Selvakumar, Krishnan Vasanth

**Affiliations:** 1 Molecular Biology Laboratory, Department of Botany, School of Life Sciences, Bharathiar University, Coimbatore, Tamil Nadu 641046 India; 2 PG Research Department of Botany, Kongunadu Arts and Science College, Bharathiar University, Coimbatore, Tamil Nadu 641029 India; 3 Department of Biochemistry, School of Life Sciences, Bharathiar University, Coimbatore, Tamil Nadu 641046 India

**Keywords:** Horse gram, *In planta* transformation, Optical cell density, Sonication, Vacuum infiltration, GUS expression

## Abstract

**Background:**

Recalcitrant nature is a major constraint for the in vitro regeneration and genetic transformation of leguminous species members. Therefore, an improved genetic transformation in horse gram has been developed via *in planta* method, in which *Agrobacterium* strain harboring binary vector pCAMBIA2301 was used for the transformation. Several factors affecting *in planta* transformations were put forth viz. *Agrobacterium* cell density, co-cultivation, and sonication combined with vacuum infiltration duration which were optimized.

**Results:**

Germinated seeds were sonicated and vacuum infiltrated with different densities of *Agrobacterium* culture and co-cultivated in half-strength MS medium with 100 μM of acetosyringone for 48 h. Seedlings were washed with cefotaxime and sowed in vermiculite soil for maturation. T_1_ plants were subjected to histochemical and molecular analysis to ensure transformation efficiency. Among various combinations analyzed, maximum transformation efficiency (20.8%) was attained with seeds of 5 min sonication combined with vacuum infiltration with 0.6 optical density of *Agrobacterium* culture.

**Conclusions:**

It concludes that a different *Agrobacterium* cell density with sonication combined with vacuum infiltration has improved transgenic efficiency in horse gram plants. This simple and efficient method is feasible for the stable expression of foreign genes that could be beneficial for future food security.

## Background

Horse gram (*Macrotyloma uniflorum* Lam. Verdc.) belongs to the family member of Fabaceae, widely used as a food crop particularly in Asian countries like Sri Lanka, Burma, and India [1]. In India, it is mainly cultivated in dryland areas of 3.25 lakh hectares that accounts for over 90% in Tamil Nadu and Andhra Pradesh state [2], although it is susceptible to numerous biotic and abiotic factors such as yellow mosaic disease, anthracnose, leaf spot, rust and root rot [3–5]. The high drought-resistant characteristic makes an ideal choice for its cultivation under various climatic and edaphic conditions which provides a possible food source for the future. The less expensive source for higher protein, carbohydrate, and fiber content with lower levels of lipids makes a better choice for the developing countries [6–8]. It also has a good source of minerals and vitamins that help in weight reduction [9, 10]. Apart from its various nutraceutical properties, it gives a potential remedy for various treatments like antihyperglycemia, antiobesity, and cardiovascular diseases [11–13].

The estimated genome size of 400 Mb evolved in the active adaption and basal response against diseases and pest resistance, and it makes as an agriculturally attractive crop [14, 15]. There are several conventional breeding methods adopted to develop hybrid varieties with disease and pest resistance. Significant success was achieved in the transformation of forage and pasture legumes via *Agrobacterium*-mediated transformation, which is the most popular and efficient method of plant genetic engineering [16–19]. In this study, the “*in planta*” transformation method was studied, where *Agrobacterium* is used to infect the seedlings. This method is simple and easy to perform in less time and does not require any skilled labor. Apart from that, several new strategies have been applied via agroinfiltration, sonication, vacuum infiltration, and floral dip methods which are now gaining much importance. Recently, several reports on the *in planta* transformation methods are emerging more than *in vitro* culture techniques [20–23].

Previous reports reveal inadequate information regarding the transformation in horse gram. Hence, the present research work has been carried out to produce the transgenic horse gram by *in planta* method optimized with different *Agrobacterium* optical cell densities and sonication combined with vacuum infiltration.

## Methods

### Collection of seeds and sterilization

Seeds of horse gram var. Paiyur 1 and Paiyur 2 were purchased from Regional Research Station, Paiyur, Tamil Nadu, India. Initially, the seeds were washed with Tween 20 solution for 5 min and rinsed using running tap water. Surface sterilization was done with 4% sodium hypochlorite for 5 min, followed by 70% ethanol washes for 2 min. Further, the seeds were properly washed thrice in sterile double-distilled water followed by surface sterilization with 0.1% HgCl_2_ (w/v) for 5 min. Finally, the seeds were thoroughly washed for five to six times in sterile water and germinated on Whatman filter papers.

### Analysis of seed germination percentage

Seed germination refers to the initial appearance of radicle length, and nearly 2 mm was measured. To determine the germination percentage, healthy seeds were selected and placed in Petri plates with moistened Whatman filter papers in dark conditions at 25 ± 2 °C. Germination rate was counted every day (up to the 7th day). Germination percentage was calculated by using the following formula:

$$ \mathrm{Germination}\  percentage=\frac{\mathrm{number}\  of\ seeds\ germinated}{\mathrm{total}\  number\ of\ seeds}\times 100 $$

### Gene, strain, binary vector, and *Agrobacterium* culture

**Fig. 1:**
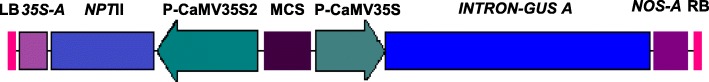
T-DNA regions of binary vector pCAMBIA2301 used for the *in planta* transformation have the *NPT*II gene as plant selectable marker in the left border (LB) driven by CaMV35S2 promoter and CaMV35S terminator and Intron–*GUS* A as a reporter gene in the right border (RB) driven by CaMV35S promoter and NOS-terminator

For transformation studies, *Agrobacterium tumefaciens* strain EHA105 harboring the binary vector pCAMBIA2301 was used. The T-DNA region carries *uid*A or *GUS*–*A* reporter gene and was initiated by CaMV35S promoter and NOS terminator sequences, and the kanamycin-resistant gene, *NPT*II, was governed by the CaMV35S promoter and terminator sequences (Fig. 1). A loop of a colony from the mother culture was inoculated in a 50 mL of yeast extract mannitol (YEM) medium supplemented with kanamycin 50 mg/L and rifampicin 20 mg/L for 16–18 h at 28 °C with 180 rpm. The cells of 200 μL culture at log phase were taken and added into a fresh 100-mL medium containing the above antibiotics at 28 °C. Afterwards, the *Agrobacterium* culture with various cell densities of 0, 0.3, 0.6, and 0.8 OD was harvested by centrifugation at 8000 rpm for 10 min. The supernatants were discarded and the resulting pellet was dissolved in an infiltration medium comprising 3 mM MES, 1/2 strength MS medium supplemented with 3% sucrose, and 100 μM acetosyringone (final pH 5.4).

### *In planta* transformation

**Fig. 2:**
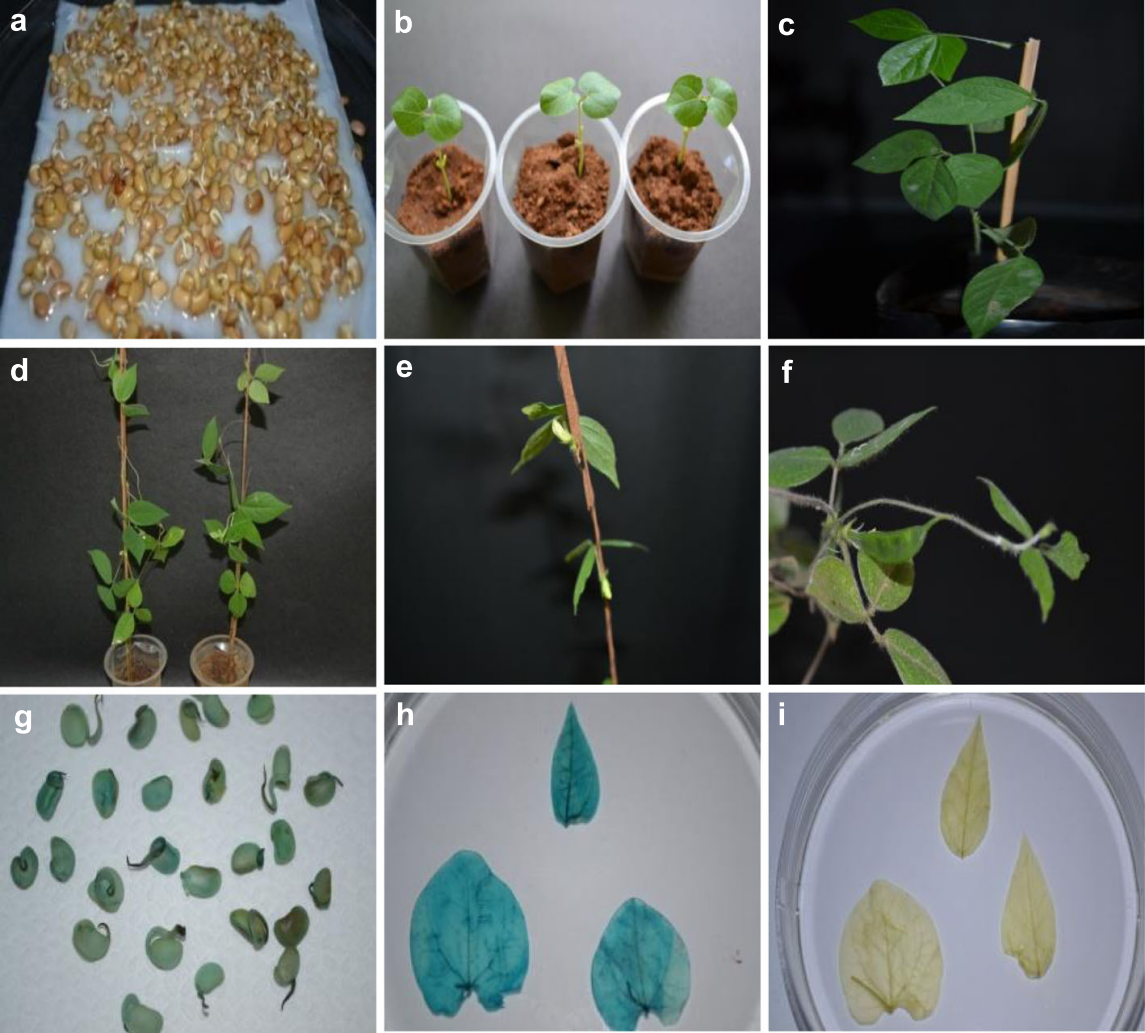
*In planta Agrobacterium*-mediated genetic transformation of horse gram (var. Paiyur 2). **a** Germinated horse gram seeds used for the infection of *Agrobacterium tumefaciens* strain. **b** 7-day-old acclimatized putatively transformed plant. **c** 14-day-old acclimatized putatively transformed plant. **d** 50-day-old acclimatized putatively transformed plant by seedlings co-cultivated with *Agrobacterium tumefaciens* strain EHA105 harboring pCAMBIA2301 binary plasmid. **e** Putatively transformed plants shows the flowering. **f** Pod formation at 55 days. **g** GUS histochemical staining was performed in *T*_1_ horse gram seedlings. **h** Leaves show GUS expression. **i** Non-transferred (control) plant leaves show no GUS expression at all

The horse gram, var. Paiyur 2, was used to study the *in planta* transformation efficiency. Several factors were optimized such as *Agrobacterium* cell density, infection time, and sonication combined vacuum infiltration. Initially, seeds were surface-sterilized and germinated for 24 h in dark condition on sterilized moistened paper towels in Petri dishes at 25 °C. (Fig. 2a). Plumules of 1 cm in length devoid of seed coats were used for transformation. *Agrobacterium* cells were inoculated in the YEM liquid medium with respective antibiotics and cultured overnight at 28 °C with 180 rpm. *Agrobacterium* cells were harvested at different cell densities and resuspended in a 50-mL infiltration medium containing 100 μM acetosyringone.

The seeds were gently pricked two to three times using a sterile needle and immediately transferred into the *Agrobacterium* infiltration medium at different time (0, 10, 15, and 30 min) intervals followed by sonication for 0, 2.5, 5.0, and 7.5 min (temperature 27 °C; 8 s on, 2 s off; 17% power) by using an ultrasonic homogenizer (SKL–150 DN model, frequency 45 kHz). Further, sonicated seeds were placed in a desiccator and vacuum infiltrated for 0, 2.5, 5.0, and 7.5 min (300 mmHg) using a vacuum pump instrument (GAST DOA–P704–AA). Subsequently, seeds were blotted on a sterile filter paper for 15 min for the removal of excess *Agrobacterium* cells. The seeds were further transferred to Petri dishes for co-cultivation in the dark at 25 °C for 48 h. The co-cultivated seeds have been rinsed thrice in sterile double-distilled water supplemented with 250 mg/L of cefotaxime to remove *Agrobacterium* contamination. The seedlings were transferred to the pots containing autoclaved vermiculite soil for further germination and maturity (Fig. 2b–f) whereas the seeds without *Agroinfection* were used as control. The seeds were collected from agroinfected *T*_0_ plants and germinated on the half-strength MS medium supplemented with 100 mg/L kanamycin for the screening of putative transgenic plants. Subsequently, the *T*_1_ plants were used for the GUS histochemical staining assay and molecular confirmation.

### GUS histochemical analysis for stable gene expression

This method was carried out to analyze the stable *GUS* gene expression in *T*_1_ germinated seedlings and leaf samples according to the described procedure [24]. Initially, the seedlings and leaf samples were washed in 50 mM sodium phosphate buffer (pH 7.0) and incubated in a GUS histochemical reagent (50 mM sodium phosphate buffer (pH 7.0), 0.5 M EDTA (pH 7.0), 100 mM K3[Fe(CN)6], 100 mM K4[Fe(CN)6], 1% Triton X–100) for 30–45 min at 37 °C. Afterwards, the samples were transferred into a X-gluc staining solution (2 mM X-gluc, 50 mM sodium phosphate buffer, 20% methanol) and placed in dark condition for 24–48 h at 37 °C. After incubation, the chlorophyll pigments were completely destained with 100% ethanol and the samples were observed for the GUS expression.

### Isolation of plant genomic DNA and PCR analysis

Plant genomic DNA was isolated from leaf samples of selected *T*_1_ GUS-positive and wild-type control plants using a modified cetyltrimethylammonium bromide (CTAB) method [25]. Polymerase chain reaction (PCR) was executed to find the presence and integration of both *GUS* and *NPT*II gene fragments using genomic DNA isolated from *T*_1_ transgenic and non-transgenic (negative control) leaf samples along with plasmid pCAMBIA2301 control (positive control). The standard PCR procedure was followed using primers specific to the *NPTII* gene (FP: 5′-TCAGAAGAACTCGTCAAGAAGGCGATA-3′; RP: 5′-GGGGATTGAACAAGATGGATTGCACGC-3′) and *GUS* gene (FP: 5′-TTATGCGGGCAACGTCTGGTATCA-3′; RP: 5′-ACGCTTGGGTGGTTTTTGTCA-3′). The amplification of respective genes was performed in a PCR thermal cycler–200^TM^ (MJ Research Inc., Waltham, Mass, USA). The PCR conditions were programmed with 94 °C for 5 min of initial denaturation, repeated for 32 cycles at 94 °C for 1 min of denaturation, 60 °C for 1 min of annealing, and 72 °C for 1 min of extension, followed by 72 °C for 10 min of final extension. Finally, the amplified PCR fragments were confirmed qualitatively by electrophoresis in 0.8% agarose gel and gel image captured using a Syngene gel documentation system (G-Box).

### Total RNA isolation and semi-quantitative RT-PCR analysis

Total RNA was extracted from GUS and genomic PCR-positive transgenic and wild-type control plants by using spectrum^TM^ plant total RNA isolation kit (Sigma-Aldrich, USA). Semi-quantitative reverse transcriptase polymerase chain reaction (RT-PCR) was performed to study the stable gene expression of *NPT*II and *GUS* transcripts. One microgram of total RNA was reverse-transcribed to single-stranded cDNA using AffinityScript QPCR cDNA Synthesis Kit according to the manufacturer’s guidelines (Stratagene, USA). PCR cycling parameters were set as initial denaturation of 94 °C for 3 min, repeated by 30 cycles of 94 °C for 40 s, 60 °C for 40 s, and 72 °C for 60 s, and a final extension at 72 °C for 10 min. Amplified fragments were analyzed by 0.8% agarose gel electrophoresis and then visualized using Syngene gel documentation system (G-Box).

### Statistical analysis

The data were analyzed using SPSS software (Version 17.0, SPSS Inc, USA). The experiments were performed thrice and subjected to one-way analysis of variance (ANOVA) followed by Duncan’s multiple range test (DMRT) at *P* < 0.05 significance level.

## Results

**Fig. 3:**
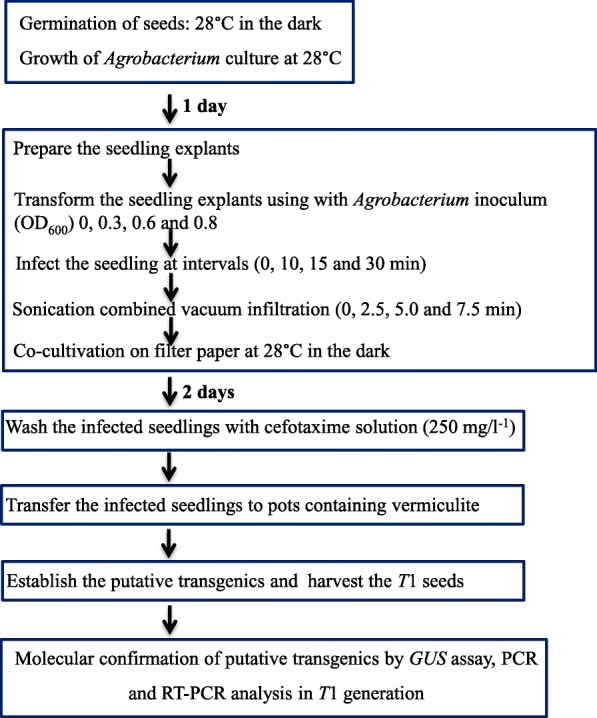
Flow chart of *Agrobacterium*-mediated *in planta* transformation protocol for the development of transgenic horse gram plants

The present study describes a rapid and efficient protocol for the development of transgenic horse gram as an alternative for tissue culture method (Fig. 3).

### Assessment of seed germination percentage

The determination of seed germination percentage provides importance for the *in planta* transformation to obtain maximum transgenic plants. The seeds of both Paiyur 1 and Paiyur 2 variety showed 87 and 93% germination, respectively. Among these two varieties, Paiyur 2 exhibited maximum germination percentage (93%). Therefore, further studies were carried out in the Paiyur 2 variety to assess different parameters influencing the *in planta* method.

### *Agrobacterium* cell density on survival rate and transgenic efficiency

**Fig. 4:**
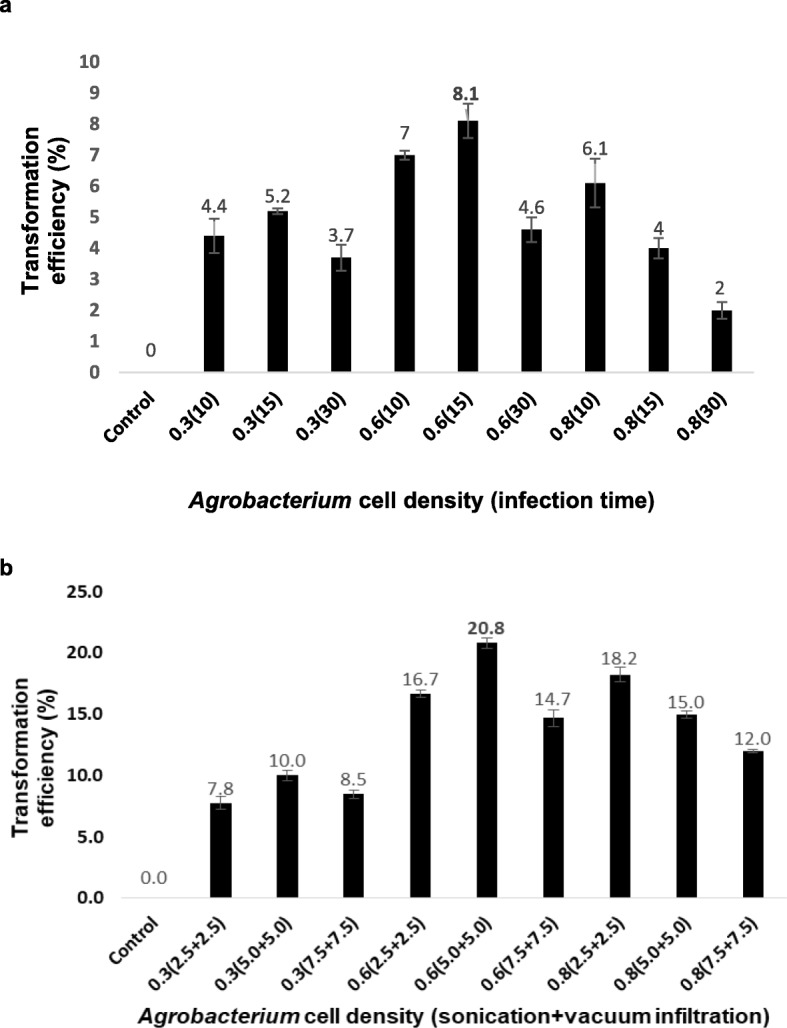
Effect of *Agrobacterium* optical cell density, infection period, and sonication combined with vacuum infiltration on influencing the *in planta* transformation of horse gram. **a** Effect of *Agrobacterium* cell density and infection time on transformation efficiency. **b** Effect of *Agrobacterium* cell density and sonication combined with vacuum infiltration on influencing the *in planta* transformation efficiency

*Agrobacterium* optical cell density and infection time plays a significant role in the *in planta Agrobacterium*-mediated transformation. Survivability index was the crucial parameter to achieve the maximum number of transgenic plants. Correspondingly, different growth phases of *Agrobacterium* (0, 0.3, 0.6, and 0.8 OD) culture with different infection periods (0, 10, 15, and 30 min) were tested to obtain the maximum plants’ survival. Among different combinations tested, the maximum percent of plant survivability (83.3%) was obtained at 0.3 OD with 10 min of infection period whereas the least survival (40%) was recorded at 0.8 OD with 30 min of infection period (Fig. 4a). Among various *Agrobacterium* cell density and infection times tested, seeds were subjected to 0.6 OD with 15 min infection time resulted in improved transformation efficiency of 8.1% (Table 1). Further, 0.8 OD with 30 min infection time has resulted in a significant reduction in transformation efficiency.

**Table 1:** Effects of different *Agrobacterium* culture densities and infection period on the survival rate and *gus A* gene expression in horse gram plants

*Agrobacterium* optical cell density (OD)	Infection time (min)	No. of seeds infected	Mean no. of survived plants (%)	Mean no. of plants expressing *gus A* gene
Control	0	100	93.33 ± 0.72^a^	0 ± 0^f^
0.3	10	100	83.33 ± 0.42^b^	4.4 ± 0.55^d^
	15	100	73.67 ± 1.14^d^	5.2 ± 0.09^cd^
	30	100	60.67 ± 0.56^f^	3.7 ± 0.42^d^
0.6	10	100	81.33 ± 0.67^bc^	7.0 ± 0.15^ab^
	15	100	78.33 ± 0.83^c^	8.1 ± 0.55^a^
	30	100	70.00 ± 0.77^de^	4.6 ± 0.40^cd^
0.8	10	100	66.33 ± 0.79^e^	6.1 ± 0.79^bc^
	15	100	50.00 ± 0.77^g^	4.0 ± 0.33^d^
	30	100	40.00 ± 0.58^h^	2.0 ± 0.27^e^

Mean values of three separate trials (±) with standard errors. In each column, numbers with different letters indicate they are considerably different from each other according to Duncan’s multiple range test at a probability level of 5%

### Influence of sonication combined with vacuum infiltration on transgenic efficiency

To pinpoint the efficient methods for transformation, different *Agrobacterium* cell densities (0, 0.3, 0.6, and 0.8 OD) with different time intervals of sonication and vacuum infiltration (0, 2.5, 5.0, and 7.5 min) were recorded. Among different combinations tested, the highest transformation efficiency (20.8%) was obtained when seeds were subjected 5 min of sonication and vacuum infiltration with 0.6 optical density of *Agrobacterium* culture. However, sonication and vacuum infiltration beyond 5 min has reduced the transformation efficiency (Table 2 and Fig. 4b). Similarly, transgenic recovery was also decreased while increasing the cell density. The effect of sonication and vacuum infiltration has many disadvantages on the survival rate of germination; however, putative transgenic efficiency of the combination was the maximum.

**Table 2:** Effects of different *Agrobacterium* culture densities and duration of sonication + vacuum infiltration on the survival rate and *gus A* gene expression in horse gram

*Agrobacterium* cell density (OD)	Sonication + vacuum (min)	No. of infected seeds	Mean no. of survived plants (%)	Mean no. of plants expressing *gus A* gene
Control	0	100	94.00 ± 0.58^a^	0 ± 0^h^
0.3	2.5 + 2.5	100	87.33 ± 0.56^b^	7.8 ± 0.51^g^
	5.0 + 5.0	100	82.67 ± 0.76^cd^	10.0 ± 0.42^f^
	7.5 + 7.5	100	68.33 ± 0.95^ef^	8.5 ± 0.32^g^
0.6	2.5 + 2.5	100	85.00 ± 0.76^bc^	16.7 ± 0.31^c^
	5.0 + 5.0	100	80.00 ± 0.52^d^	20.8 ± 0.40^a^
	7.5 + 7.5	100	72.33 ± 0.70^e^	14.7 ± 0.69^d^
0.8	2.5 + 2.5	100	70.00 ± 0.82^ef^	18.2 ± 0.61^b^
	5.0 + 5.0	100	65.67 ± 0.75^f^	15.0 ± 0.27^d^
	7.5 + 7.5	100	52.00 ± 0.77^g^	12.0 ± 0.14^e^

Mean values of three separate trials (±) with standard errors. In each column, numbers with different letters indicate they are considerably different from each other according to Duncan’s multiple range test at a probability level of 5%

### GUS histochemical analysis of putative transgenics

GUS histochemical assay was used to analyze the *GUS* gene expression in putative transgenic horse gram plants. The assay was performed in *T*_1_ leaves and seedlings of putative transformed and control plants. An intense blue color formation was observed in the transgenic seedlings and leaf samples (Fig. 2g, h). Samples devoid by the blue color were the non-transgenics (Fig. 2i). The degree of GUS expression was not uniform in all transgenic lines analyzed.

### Molecular analysis of transgenic horse gram

**Fig. 5:**
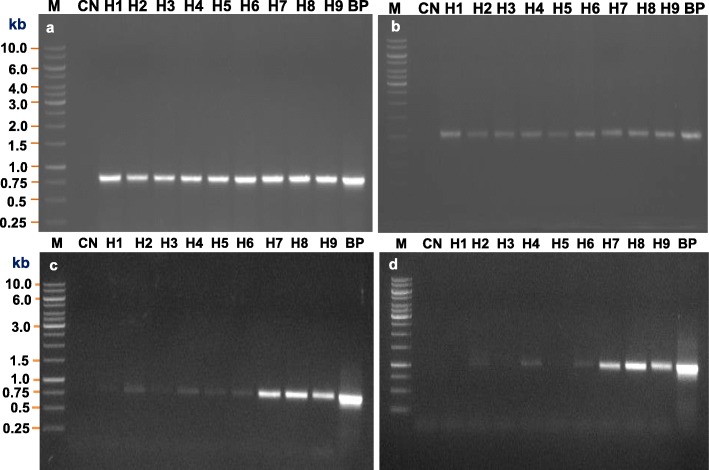
Molecular analysis of *in planta T*_1_ transgenic horse gram plants. **a**, **b** Genomic DNA was isolated from control (CN) and nine putative *T*_1_ transgenic horse gram leaf samples and the PCR analysis performed using *NPT*II (**a**) and *GUS* (**b**) gene-specific primers, respectively. **c**, **d** RT-PCR analysis of transgenic horse gram plants using *NPT*II **c** and *GUS***d** gene-specific primers, respectively. Total RNA was isolated from control and putative transgenic lines and then converted to the single-stranded cDNA and performed the RT-PCR to study the expression of *NPT*II and *GUS* transcript. Note: 796 bp and 1.0 kb PCR products were observed in all transgenic plants except control (CN) using *NPT*II and *GUS* gene-specific primer, respectively. *M* 1.0 kb DNA ladder (Fermentas, Thermo Scientific), *CN* control plant, *H1–H9* different transgenic lines, *BP E. coli* binary plasmid pCAMBIA2301

The presence and expression of the transgene in transgenic horse gram plants were confirmed by genomic PCR and semi-quantitative RT-PCR analysis, respectively. Genomic DNA from nine GUS-positive transgenic (H1, H2, H3, H4, H5, H6, H7, H8, and H9) and non-transformed control (WT) plants were isolated and PCR was performed using *NPT*II- and *GUS-*specific primers. The PCR yielded an amplicon size of 796 bp and 1.0 kb of the *NPT*II (Fig. 5a) and *GUS* gene (Fig. 5b) fragments, respectively, in all GUS-positive transgenic lines and also in DNA from plasmid control were used as a positive control, whereas amplification fragments were absent in a non-trangenic wild-type control (WT) plant which was used as a negative control (Fig. 5a, b).

To analyze the expression of the transgene, semi-quantitative RT-PCR analysis was performed in nine (H1, H2, H3, H4, H5, H6, H7, H8, and H9) GUS- and PCR-positive plants. The RT-PCR yielded a fragment of 796 bp and 1.0 kb of the *NPT*II (Fig. 5c) and *GUS* (Fig. 5d) transcript, respectively, in all transgenic lines and also in plasmid control which was used as positive control and absent in a non-trangenic control (WT) plant which was used as negative control (Fig. 5c, d).

## Discussion

The agricultural revolution has been happening predominantly due to the remarkable nature of seeds for its convenient storage and fast propagation and makes it ideal for human requirements. Horse gram is one of the prehistoric pulse crops from the time when agriculture particularly started in South Asia [2, 26]. Among different pulse crops, the cultivation and exploration of this crop become limited due to wild dormancy and various biotic factors. In this study, a simple and effective *in planta Agrobacterium*-mediated transformation method for the development of transgenic horse gram has been reported. Here, seeds were used as an explant for the transformation because of its fast-multiplying cells during germination which is useful for the integration of the target gene. The seed germination percentage was best in Paiyur 2 variety compared to Paiyur 1. The thick and hard seed coats of horse gram is one of the major drawbacks for the efficient uptake of foreign DNA into the plant genome, even though numerous methods can be adopted for the efficient recovery of transgenic plants. There are several recent reports on the seeds as explants for the *in planta* transformation [27–30].

Selection of appropriate strain and cell density are the prerequisites for transformation in dicot species. In several leguminous members, the *Agrobacterium* EHA105 strain was found the best choice for the transformation recovery and stable integration of genes [31–33]. Based on our investigation, an *Agrobacterium* cell density of 0.6 OD produced the highest number of GUS positive in both sonication combined vacuum treatment. However, the percentage of survival rate was gradually decreased in proportional to cell density and infection time. We found that some plants underwent necrosis and death, while the duration of infection and the cell density becomes increased. Similarly, infection time > 10 min contributed to *Agrobacterium* resulted in overgrowth and > 35 min resulted in the browning of the target tissue. Correspondingly, nominal cell density with increased co-cultivation duration has the lowest stress response with maximum survivability of explants [21, 34, 35]. On the contrary, herbicide-resistant wheat and cotton plants raised by *in planta* method resulted in a maximum survival rate with 3.07% and 0.12% transformation efficiency [30, 36].

Nowadays, sonication and vacuum infiltration are the most convenient and simple method adopted for the *in planta* transformation. Improvising the penetration of *Agrobacterium* cells into the plant genome by the influence of sonic waves and vacuum tends to increase the efficiency of gene transfer. The purpose of vacuum infiltration after sonication may offer an additional access point for *Agrobacterium*, making it easier to integrate the transformation for the tissues [37]. Soybean cotyledonary node explants subjected to 2 min of sonication followed by vacuum infiltration resulted in 18.6% transformation efficiency [38], whereas precultured peanut explants are subjected to 6 min of sonication and 3 min of vacuum infiltration in *Agrobacterium* suspension resulting in 31.3% transformation efficiency [28]. The combined effects of sonication and vacuum infiltration treatments had been reported to have increased transformation efficiency in kidney bean and persimmon [39, 40].

For precise screening of transgenic plants, the *GUS* reporter gene was used to evaluate the promoter function for the visualization of expressive patterns throughout the plant tissues [41, 42]. In the present study, there was a high level of GUS expression observed in leaf materials than stem and seed explants. Correspondingly, blue color visualization was absent in control (non-transformed) plants. Different parameters like *Agrobacterium* cell density, sonication, vacuum infiltration, and co-cultivation were influenced by the transformation efficiency and GUS expression in transgenic plants. This may be due to the integration, copy number, and expression of the transgene in plants [43, 44] or due to the methylation of the chromosomal integration region [45]. Finally, the stable expression and integration of desired genes were confirmed by PCR and semi-quantitative RT-PCR.

## Conclusions

The present study demonstrated the development of transgenic horse gram by subjecting *in planta* transformation protocol. The results revealed that the percentage of survival rate was gradually decreased with increased *Agrobacterium* cell density and infection period. The combined effects of sonication and vacuum infiltration showed greater transgenic efficiency, but the lowest survival rate of germinating seeds was observed. Germinated seedlings of 24 h were used for the infection of *Agrobacterium* culture at 0.6 OD followed by 5 min of sonication and vacuum (300 mmHg) infiltration followed by 2 days of co-cultivation resulted in 20.8% of the transgenic efficiency. *Agrobacterium*-mediated genetic transformation by *in planta* method in horse gram is the first report that states this kind. This protocol would be efficient and easier for genetic manipulation against several biotic factors and also helps to improve the nutritional quality of the pulse crop for human welfare.

## Data Availability

Not applicable

## Abbreviations

OD: Optical density
CaMV: Cauliflower mosaic virus
CTAB: Cetyltrimethylammonium bromide
T-DNA: Transfer-DNA
HgCl_2_: Mercuric chloride
MES: 2-(N-morpholino)ethanesulfonic acid
GUS: β-Glucuronidase
X-gluc: 5-Bromo-4-chloro-3-indolyl-beta-d-glucuronic acid
EDTA: Ethylenediaminetetraacetic acid
*NPT*II: Neomycin phosphotransferase
RT-PCR: Reverse transcription polymerase chain reaction
ANOVA: Analysis of variance
DMRT: Duncan’s multiple range test
